# No effect of transcutaneous auricular vagus nerve stimulation on response inhibition

**DOI:** 10.3389/fnhum.2026.1742058

**Published:** 2026-05-07

**Authors:** Shunpei Yamamoto, Shota Miyaguchi, Kyosuke Shiga, Hirotake Yokota, Yasuto Inukai, Naofumi Otsuru, Hideaki Onishi

**Affiliations:** 1 Graduate School, Niigata University of Health and Welfare, Niigata, Japan; 2 Institute for Human Movement and Medical Sciences, Niigata University of Health and Welfare, Niigata, Japan; 3 Department of Physical Therapy, Niigata University of Health and Welfare, Niigata, Japan; 4 Department of Rehabilitation, St. Marianna University Hospital, Kawasaki, Kanagawa, Japan

**Keywords:** noradrenaline, response inhibition, salivary alpha-amylase, stop signal task, transcutaneous auricular vagus nerve stimulation

## Abstract

**Introduction:**

Response inhibition, which stops an ongoing action, is important for daily life activities. Reportedly, noradrenaline (NA), a neurotransmitter, is involved in response inhibition. In recent years, transcutaneous auricular vagus nerve stimulation (taVNS) has gained attention for its ability to induce NA release in various areas of the brain. Thus, taVNS can potentially improve response inhibition; however, this remains unverified.

**Methods:**

The present study investigated the effects of taVNS on response inhibition by measuring salivary alpha-amylase (sAA), an indirect indicator of NA. This study implemented a repeated-measures pre–post approach across conditions, with 24 healthy men (age, 21.3 ± 1.6 years) performing a stop signal task and undergoing sAA measurement before and after the taVNS intervention. taVNS was performed for 30 min at an intensity midway between the sensory and pain thresholds. Participants underwent two stimulation conditions, namely taVNS targeting the left concha and sham stimulation targeting the left earlobe, in a randomized order. Response inhibition was evaluated by comparing the stop signal reaction time (SSRT) before and after taVNS intervention.

**Results:**

Under taVNS, sAA levels significantly increased after the stimulation. However, no significant difference in SSRT was observed between before and after the intervention under either condition.

**Discussion:**

These results suggest that taVNS increases sAA levels after stimulation; however, it does not influence response inhibition, suggesting that the modulation of NA by taVNS is insufficient to improve response inhibition.

## 1 Introduction

The ability to stop physical activity in response to changes in the external environment, termed response inhibition, is frequently required in daily life. This process can be classified into two forms, namely proactive and reactive response inhibition (Hermans et al., 2017; Rawji et al., 2020; Schachar et al., 2007). Proactive response inhibition involves suppressing a motor response before a movement begins, whereas reactive response inhibition refers to suppressing a movement that is already underway. In particular, reactive response inhibition is frequently required in daily life to facilitate appropriate behavioral adjustments to changes in the external environment and to prevent collisions with other individuals or obstacles during ongoing activities. Impairments in this inhibitory function may compromise safe and adaptive behavior.

One method designed to assess reactive inhibition is the stop signal task (SST) (Verbruggen and Logan, 2008). In this task, a button-press response is executed in response to a repeatedly presented “go” signal, but a “stop” signal is presented at certain probabilities to suppress the button-press response. This task permits estimation of the stop signal reaction time (SSRT), which represents the time required for response inhibition (Sarkar et al., 2020). The SST is reportedly influenced by the neurotransmitter noradrenaline (NA), which plays an important role in suppressing responses (Eagle et al., 2008). Response inhibition is impaired in conditions such as attention-deficit/hyperactivity disorder (ADHD), obsessive-compulsive disorder, eating disorders, and substance dependence (Bartholdy et al., 2016; Lipszyc and Schachar, 2010; Smith et al., 2014). In particular, dysregulation of the noradrenergic network is believed to underlie the symptoms of ADHD (Noah and Sedky, 2025). It has been reported that SSRT is shortened by the administration of atomoxetine, a selective NA reuptake inhibitor, to patients with ADHD (Chamberlain et al., 2007), and similar effects have been observed in healthy participants (Chamberlain et al., 2006). Atomoxetine is thought to increase extracellular NA levels in the central nervous system, thereby enhancing synaptic transmission efficiency and potentially contributing to inhibitory control. However, the relationship between noradrenergic modulation and inhibitory control is not deterministic but rather probabilistic, and may depend on various contextual factors, including task structure, cognitive load, and individual differences.

Transcutaneous auricular vagus nerve stimulation (taVNS) is a non-invasive technique that stimulates the auricular branch of the vagus nerve and has been proposed as a non-invasive approach distinct from cervical implanted VNS (Toffa et al., 2020). taVNS has attracted increasing attention due to its reported therapeutic effects across a wide range of clinical conditions, including epilepsy (Toffa et al., 2020), stroke (Badran et al., 2023), depression (Guo et al., 2024), chronic pain (Napadow et al., 2012), COVID-19 (Boezaart and Botha, 2022), and cardiac arrhythmias (Stavrakis et al., 2020); however, the precise neural mechanisms underlying these effects remain insufficiently understood. One of the mechanisms underlying the effects of taVNS is believed to involve activation of the locus coeruleus–noradrenergic (LC-NA) system. Approximately 80% of the vagus nerve consists of afferent fibers, and the afferent inputs project to the nucleus tractus solitarius (NTS) and the locus coeruleus (LC) (Nemeroff et al., 2006; Ruffoli et al., 2011). The LC contains noradrenergic neurons and projects NA to a wide portion of the central nervous system (Benarroch, 2018; Hachem et al., 2018; Ma et al., 2023). The influence of taVNS on the activity of the human LC–NA system has been investigated in several studies. Using functional magnetic resonance imaging (fMRI), previous human studies have reported activation of the brainstem, including the LC, following taVNS (Frangos et al., 2015; Yakunina et al., 2017). Converging evidence is provided by animal studies employing invasive vagus nerve stimulation, which have demonstrated increases in extracellular NA concentrations in the central nervous system (Raedt et al., 2011; Roosevelt et al., 2006). Recent studies utilized several physiological findings as indirect evaluation markers of the LC-NA system. Among these measures, salivary alpha-amylase (sAA) has been widely used as an indirect marker of noradrenergic activity, supported by pharmacological studies demonstrating associations between central NA signaling and sAA levels (Ehlert et al., 2006; Warren et al., 2017). Previous studies have reported increases in sAA following taVNS (Ventura-Bort et al., 2018), and sAA has been proposed to reflect taVNS-related changes in noradrenergic activity (Warren et al., 2019; Giraudier et al., 2022). Based on these findings, taVNS has attracted attention as a potential neuromodulatory approach for influencing the LC–NA system, with sAA serving as an indirect index of stimulation-related noradrenergic modulation.

Several prior studies examined the effects of taVNS on cognitive control. Previous studies reported improvements in associative memory during taVNS (Jacobs et al., 2015) and enhancements in response selection during continuous movements (Jongkees et al., 2018). In recent years, the effects of taVNS on inhibitory function have received increasing attention. For example, taVNS was reported to enhance conflict adaptation (Fischer et al., 2018). Another study observed improved inhibitory control performance under working memory load (Beste et al., 2016). More recent electrophysiological evidence suggests that taVNS may modulate neural processes underlying inhibitory control during response inhibition tasks (Pihlaja et al., 2020). However, the direction and behavioral consequences of such neuromodulatory effects do not appear to be uniform. For example, another study demonstrated that taVNS modulated frontal alpha-band activity during a conflict monitoring task, reflecting reduced inhibitory gating of interfering information, even though behavioral performance was not consistently improved (Konjusha et al., 2022). These reports suggest that the regulation of NA during taVNS contributes to cognitive control and inhibitory functions. Although many reports described the effects of NA regulation during taVNS on cognitive control and inhibitory function, few studies assessed the aftereffects of taVNS, and knowledge in this area remains limited. Neuromodulatory systems such as the LC–NA system are known to exhibit both phasic and tonic modes of activity (Aston-Jones and Cohen, 2005), and stimulation-induced changes in tonic noradrenergic activity may persist beyond the stimulation period. Ventura-Bort et al. (2018) reported an increase in sAA following taVNS, suggesting an enhancement of NA activity. Although it remains unclear to what extent and for how long indirect indicators like sAA are maintained after taVNS in humans, the above findings indicate that taVNS may improve response inhibition even after stimulation. However, it remains uncertain whether NA regulation following taVNS contributes to this improvement. Furthermore, previous findings from non-invasive electrical neuromodulation suggest that changes in response inhibition may depend on individuals’ baseline performance (Shiga et al., 2023). However, it remains unclear whether such baseline-dependent effects also occur with taVNS. Considering these observations, baseline performance may influence the magnitude of taVNS induced improvements in inhibitory control. Together, these considerations provide a theoretical rationale for examining post-stimulation effects of taVNS on inhibitory control. Importantly, the effects of neuromodulation on inhibitory control may depend on task demands. Inhibitory performance is not determined solely by conflict processing, but is also influenced by response-selection requirements and motor complexity. Previous studies have shown that increased movement complexity and action-selection demands can modulate inhibitory control dynamics (Gálvez-García et al., 2018). Moreover, recent evidence suggests that the effects of taVNS may become more pronounced when executive demands are increased through task complexity manipulations (Mena-Chamorro et al., 2025; Gálvez-García et al., 2026). In contrast, tasks involving simple motor responses and limited response-selection demands may reduce sensitivity to detect neuromodulatory effects. Therefore, the characteristics of the task employed may critically influence the extent to which taVNS-related changes in inhibitory control can be observed.

Additionally, taVNS has been shown to affect heart rate (HR) and heart rate variability (HRV) (Clancy et al., 2014; Antonino et al., 2017). More recent studies suggest that these effects depend not only on stimulation parameters, such as frequency and current intensity, but also on individual differences, including baseline autonomic activity assessed via cardiac spectral components and sex (Yokota et al., 2022). One possible mechanism underlying these effects involves the activity of the NTS and the LC. In healthy adults, high-frequency stimulation at 100 Hz has been shown to increase activity in the NTS and LC, which was correlated with parasympathetic indices of HRV (Sclocco et al., 2020). Taken together, these findings highlight the importance of measuring ECG-derived autonomic indices in studies investigating the effects of taVNS on cognitive and inhibitory functions.

In the present study, we investigated the effects of taVNS on sAA, an indirect marker of NA activity, and on response inhibition. Based on a previous study illustrating that sAA levels increase after taVNS (Ventura-Bort et al., 2018), we hypothesized that sAA levels would increase after taVNS in the present study. Moreover, based on findings that response inhibition performance is influenced by NA (Eagle et al., 2008), we hypothesized that sAA levels would be modulated and response inhibition would be improved by taVNS. We further hypothesized that the effects of taVNS would be greater in individuals with poorer baseline response inhibition performance. In this study, electrocardiogram (ECG) measurements were conducted because taVNS has been reported to affect autonomic nervous functions, such as HR and HRV (Clancy et al., 2014; Antonino et al., 2017). If taVNS proves effective in enhancing response inhibition, this technique could contribute to the development of new treatment and rehabilitation programs for neurological disorders and related conditions.

## 2 Materials and methods

### 2.1 Participants

A total of 24 right-handed healthy men (age, 21.3 ± 1.6 years) with no history of epileptic seizures or neurological, psychiatric, or cardiac diseases participated in this study. The sample sizes were determined using G*Power 3.1.9.7, assuming a medium effect size (f) of 0.25, an alpha level of 0.05, and a statistical power (1 − *β*) of 0.80. To minimize potential variability associated with menstrual-cycle-related influences on response inhibition and autonomic nervous system activity, the study was conducted exclusively in healthy male participants (Colzato et al., 2010; Espin et al., 2019). While this approach enhances experimental control, it necessarily constrains the external validity of the findings and limits their generalizability to other populations, including females. All participants met the following criteria: no pacemaker or metal devices in the body; no alcohol or drug dependence; no daily medication; no history of smoking; and no trauma to the ear. This study was conducted with approval from the Ethics Review Committee of Niigata University of Health and Welfare (approval number: 19224-240227). In addition, this study was conducted in accordance with the Declaration of Helsinki, and written informed consent were obtained from all participants after providing sufficient explanations of the study goals and protocols.

### 2.2 taVNS

taVNS was performed using NEMOS (Cerbomed GmbH, Erlangen, Germany). The stimulation parameters used in previous studies referenced in the present study are summarized in Table 1. In the taVNS condition, the stimulation electrode was placed on the left cymba concha, which was chosen as the stimulation site because it is innervated exclusively by the afferent fibers of the vagus nerve (Peuker and Filler, 2002). Under the sham condition, the electrode was placed on the left earlobe, which has little innervation from vagus nerve afferent fibers (Peuker and Filler, 2002). This electrode configuration is consistent with that used in previous taVNS studies assessing sAA as an index of noradrenergic activity (Ventura-Bort et al., 2018; Warren et al., 2019). After cleaning the two hemispherical titanium electrodes with alcohol wipes, we applied a conductive cream to enhance conductivity. Before attaching the electrodes, the cymba conchae and earlobes were cleaned with alcohol wipes to reduce skin resistance under the electrodes. The stimulation intensity of taVNS was set at the midpoint between each participants’ sensory and pain thresholds. This approach was adopted based on previous studies showing that the auricular branch of the vagus nerve (ABVN) is involved in somatosensory processing and that setting the stimulation intensity within this range can induce increases in sAA (Ventura-Bort et al., 2018). The sensory threshold was determined by starting the stimulation at 0.1 mA and increasing the stimulation intensity in 0.1-mA increments until the participant reported the first sensation. Next, the pain threshold was determined by increasing the stimulus intensity in 0.1-mA increments until pain was reported. This procedure was repeated three times, yielding three sensory thresholds and three pain thresholds (six values in total), which were summed and averaged to determine the stimulation intensity used in the experiment. Stimulation was delivered at a frequency of 25 Hz with a pulse width of 250 μs for 30 min, using a cycle of 30 s on and 30 s off. These stimulation parameters were matched to those used in Warren et al. (2019), who reported increases in sAA during taVNS, and the stimulation duration was comparable to that used in Ventura-Bort et al. (2018). Sham stimulation was performed on the left earlobe using the same stimulation parameters applied for taVNS. During stimulation, the participants maintained a resting position in a chair. To investigate the side effects of taVNS (itching, burning sensation, headache, dizziness, nausea), each participant rated the intensity of the side effects experienced during taVNS on a seven-point scale after the stimulation ended (0 = “no sensation,” 6 = “very strong sensation”). All participants completed both conditions in a random order. An interval of at least 5 days was applied between the conditions.

**Table 1:** Stimulation parameter and references.

Parameter	Stimulation parameter	References
Laterality and sites	taVNS: left cymba concha	Ventura-Bort et al. (2018) Warren et al. (2019)
	Sham: left earlobe	
Timing	Offline effect	Ventura-Bort et al. (2018)
Intensity	Midpoint between each participants’ sensory and pain thresholds	
Frequency (pulse width)	25 Hz (200–300 μs)	
Duration	35 min	
Frequency (pulse width)	25 Hz (200–300 μs)	Warren et al. (2019)
Duty cycle	30 s on and 30 s off	

### 2.3 sAA measurement

sAA was selected as a non-invasive surrogate marker because it is responsive to sympathetic activation and is considered to indirectly reflect central noradrenergic activity; accordingly, it has been widely used in psychophysiological and taVNS studies (Nater et al., 2007; Giraudier et al., 2022). In addition, sAA allows for repeated measurements with minimal participant burden and without inducing additional stress responses. sAA levels were measured using a salivary amylase monitor (product number 59–014, Nipro Co., Ltd., Osaka, Japan). For each measurement, the paper portion of a dedicated collection tip (product number 59–010, Nipro Co., Ltd.) was placed under the tongue for approximately 40 s. The tip was then removed and visually inspected to confirm adequate saliva absorption. To reduce the influence of momentary fluctuations and potential outliers, sAA levels were assessed three consecutive times at 30-s intervals both before and after taVNS, and the median value was used for statistical analysis. sAA activity is affected by diurnal fluctuations, with levels generally increasing from afternoon to evening (Nater et al., 2007; Giraudier et al., 2022). Therefore, all experimental sessions were conducted in the afternoon. To minimize potential confounding effects of circadian variation, the timing of measurements was kept consistent across all participants and experimental conditions.

### 2.4 SST

**Figure 1:**
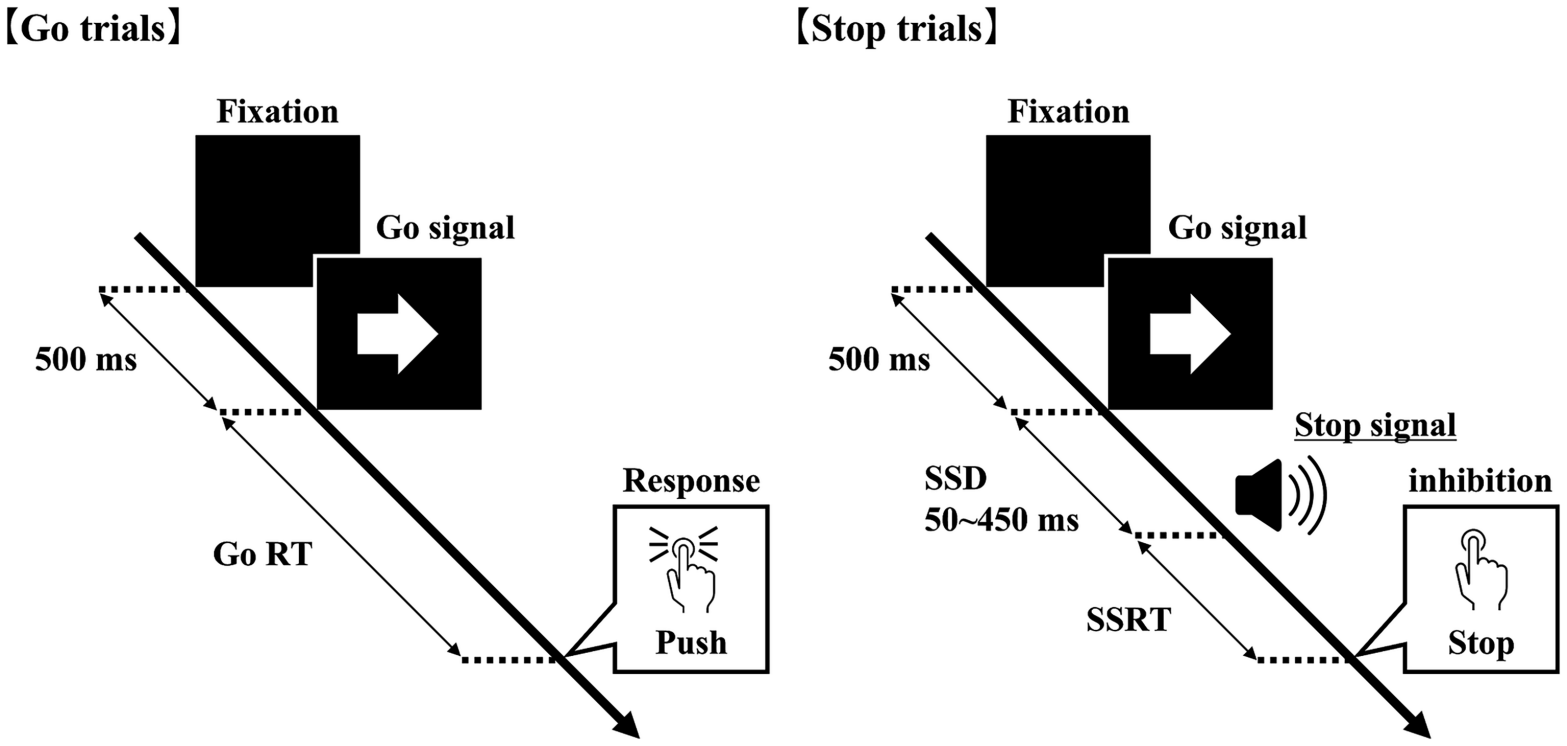
Stop signal task (SST). Go trials consisted of left and right arrows, and stop trials consisted of an auditory signal with a delay after the go signal. In the go trials, participants were instructed to press the corresponding keyboard button when a left or right arrow was presented as quickly as possible. In the stop trials, they were instructed to refrain from pressing the button. The stop signal delay (SSD) was initially set at 250 ms and adjusted based on the performance of each participant.

The SST (Inquisit 6, Millisecond Software, LLC, Seattle, WA, USA), which consists of go and stop trials, was performed as previously utilized (Shiga et al., 2023) (Figure 1). A 12.5-inch laptop and headphones were used for the SST. Participants wore headphones in a quiet room, assumed an edge-seated position, and performed the task by placing their right index finger on the “K” key and their left index finger on the “D” key of the laptop. In the go trials, participants were instructed to follow the left and right arrows (Go signals) displayed on the laptop screen and press the key corresponding to each arrow (right arrow: K, left arrow: D) as quickly and accurately as possible. In addition, in 25% of the trials (stop trials), participants were presented with a buzzer sound (stop signal) through headphones between 50 and 450 ms (stop signal delay [SSD]) after the go signal was displayed. A beep, serving as the buzzer sound, lasted roughly 75 ms. The volume was configured via the laptop’s settings and set to 15 out of 100. If a stop signal was presented after the go signal, then the participants were instructed to suppress their button-press response. The SSD was varied according to each participants’ performance, starting at 250 ms and increasing by 50 ms if the task was successful (successful response inhibition) and decreasing by 50 ms if the participant failed. In other words, the SSD was automatically adjusted for each trial so that the difficulty was increased for each successful stop trial and decreased for each unsuccessful stop trial. The SST consisted of four blocks, with the first block serving as a practice session comprising 32 trials (go trials: 24 trials, stop trials: eight trials). The subsequent three blocks were test sessions, each containing 72 trials (go trials: 54 trials, stop trials: 18 trials), giving a total of 216 trials. Using the most reliable integration method (Verbruggen et al., 2019), SSRT was calculated as follows: (1) calculate the probability (response∣signal) of responding on a stop trial; (2) calculate the distribution of the go reaction time (GoRT) across all go trials, including failed stop trials; (3) calculate the nth RT [*n* = RT distribution × *p*(response∣signal)]; and calculate SSRT by subtracting the average SSD from the nth RT (SSRT = nth RT − average SSD). A lower value indicates better response inhibition. The present study employed an adaptive SSD procedure like that used in previous taVNS studies (Wang et al., 2022). However, it differed from prior work in the use of an auditory stop signal, a restricted SSD range, and the application of the integration method for estimating SSRT, which is currently recommended as a more reliable approach.

### 2.5 ECG

ECGs were recorded as an indirect measure to assess the effects of taVNS on autonomic cardiovascular modulation. Three Ag/AgCl surface electrodes (Blue Sensor, METS, Tokyo, Japan) were placed on the left and right subclavian regions and the left costal margin to record the ECG signal, which was amplified with an amplifier (A-DL-720·140, 4ASSIST, Nicosia, Cyprus), A/D converted, and then saved on a computer using Power Lab (AD Instruments, Colorado Springs, CO, USA). ECG recordings were performed using LabChart 8 (AD Instruments) with a sampling frequency of 1 kHz and the bandpass filter set to 0.5–35 Hz. HRV Scientific (Kubios, Washington, DC, USA) was used for the subsequent analysis. HR was calculated from the RR intervals of the recorded ECG. Power spectrum analysis was performed to calculate the low-frequency component (LF, 0.04–0.15 Hz), indicating sympathetic nerve activity (including parasympathetic nerve activity), and the high-frequency component (HF, 0.15–0.40 Hz), indicating parasympathetic nerve activity. The LF/HF ratio was then calculated. A higher LF/HF ratio indicates the dominance of sympathetic nerve activity, whereas a lower value indicates the dominance of parasympathetic nerve activity (McCraty and Shaffer, 2015). ECG measurements were recorded for 2 min before the start of the experiment (baseline) and during taVNS at 0–10, 10–20, and 20–30 min.

### 2.6 Experimental procedure

**Figure 2:**
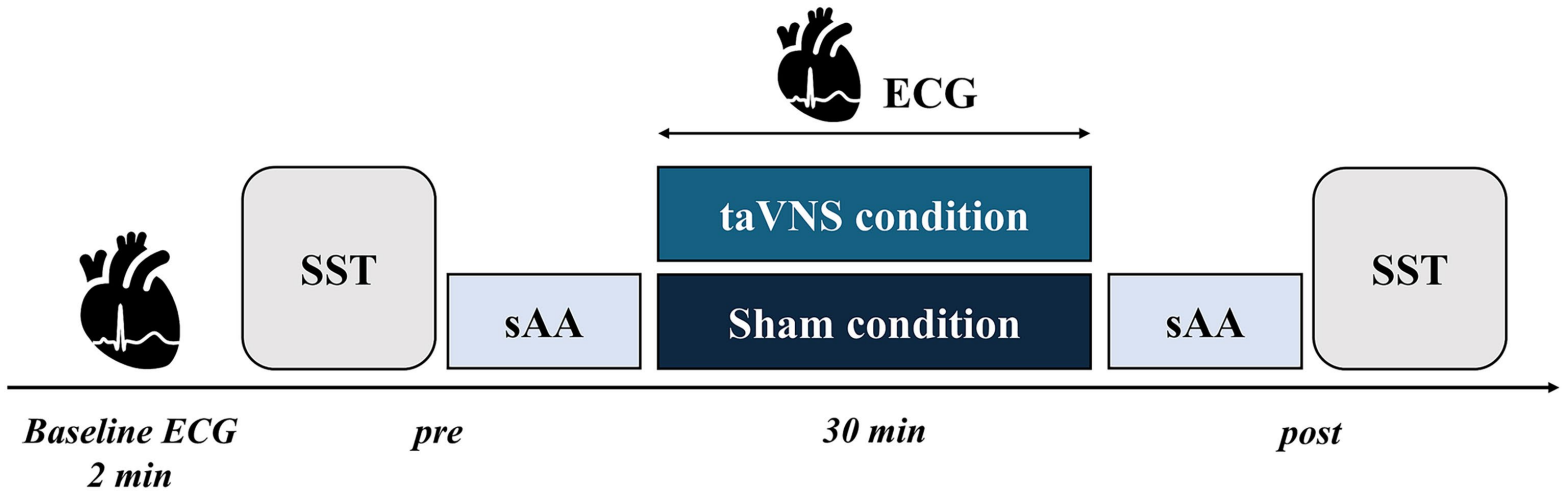
Experimental procedure. After measuring the baseline electrocardiogram for 2 min, participants performed four sets of the stop signal task (SST) before transcutaneous auricular vagus nerve stimulation (taVNS) to evaluate salivary alpha-amylase (sAA) levels (pre). Next, each condition was applied during a resting state. Afterward, sAA was evaluated again, and four more sets of SSTs were performed (post).

Figure 2 presents the experimental procedure of this study. To measure sAA, all participants were scheduled to complete the experimental procedure between 1:00 and 5:00 p.m. Participants were instructed to avoid excessive exercise and alcohol and caffeine intake the day before the experiment and to refrain from eating or drinking for 2 h on the day of the experiment. First, each participant rested in a seated position for 15 min to stabilize autonomic nervous activity. This procedure was based on a previous taVNS study conducted by our research group, in which a 20 min seated resting period was implemented prior to physiological assessments to minimize autonomic nervous system activity (Yokota et al., 2022). After this rest, each participant underwent baseline ECG. Next, SST and sAA were evaluated before the intervention (pre). Afterward, the taVNS electrodes were attached, and the stimulation intensity was determined. The stimulation was applied for 30 min, and the participants were instructed to maintain a resting seated position during the intervention. During the intervention, ECG was measured to monitor autonomic nervous system activity. After the intervention (post), the stimulation device was removed, and sAA and SST were performed again. The SST consisted of one practice set (32 trials) and three test sets (72 trials each) for pre and post which were completed within 10 min. sAA was measured three times pre- and post-taVNS, with a 30-s interval between each measurement. The total time required for the three measurements was approximately 3 min. All participants completed both conditions in a counterbalanced order, with a 1-week interval between the conditions.

### 2.7 Data and statistical analysis

GoRT, SSD, p(response∣signal), and SSRT were calculated from the SST performed before and after each stimulus. The primary outcome measure of this study was SSRT, reflecting response inhibition performance. In addition, the difference in SSRT between preand post-stimulation (ΔSSRT) was calculated as an index of change in response inhibition, with larger values indicating greater post-stimulation improvement. Secondary outcome measures included sAA, GoRT, SSD, p(response∣signal), and ECG-derived autonomic indices (HR and LF/HF). Two participants exhibited p(response|signal) values of ≥75% in the SST and were therefore excluded from all analyses, in accordance with the SST consensus guidelines, which indicate that such values reflect unreliable SSRT estimation (Verbruggen et al., 2019). For ECG analyses, HR and LF/HF were extracted as autonomic parameters. An additional three participants were excluded from the ECG analyses due to inaccurate LF/HF measurements, resulting in a final sample of 19 participants for the ECG analysis.

SPSS ver. 29 (IBM Corp., Armonk, NY, USA) was used for statistical processing. Normality was assessed using the Shapiro–Wilk test. Based on these results, Wilcoxon signed-rank tests were used to compare sAA, GoRT, SSD, and p(response∣signal) between preand post-stimulation within each condition, and the effect size r was reported. SSRT, the primary outcome measure, was analyzed using a repeated-measures two-way analysis of variance (ANOVA) to examine the main effects of time (pre, post), stimulation condition (taVNS, sham), and their interaction. Mauchly’s test of sphericity was applied, and when the assumption of sphericity was violated, Greenhouse–Geisser corrections were used. Partial η^2^ was reported as the effect size. To complement the frequentist analysis and to quantify evidence for both the alternative and null hypotheses, a Bayesian repeated-measures ANOVA was additionally conducted for SSRT, with the Bayes factor (BF_10_) used as an index of evidence strength. Regarding ECG measures, HR followed a normal distribution and was analyzed using a repeated-measures two-way ANOVA with time (baseline, 0–10 min, 10–20 min, and 20–30 min) and stimulation condition (taVNS, sham) as factors. Because LF/HF did not meet the assumption of normality, the Friedman test was applied, followed by Bonferroni-corrected *post hoc* comparisons. To test the hypothesis that individuals with lower baseline response inhibition benefit more from taVNS, the association between prestimulus SSRT (pre_SSRT) and ΔSSRT was examined using Pearson’s product–moment correlation coefficient. The significance level was set at 5% for all analyses.

## 3 Results

### 3.1 Stimulation intensity and side effects for each stimulation condition

Table 2 presents the stimulation intensity and side effects for each stimulation condition. All 22 participants received stimulation for 30 min without interruption. The stimulation intensity was significantly higher in the sham condition than in the taVNS condition (*t* (21) = −3.75, *p* = 0.001, *d* = −0.80). However, there was no significant difference between conditions regarding itching directly under the electrode (*p* = 0.197). Additionally, no participants reported experiencing a burning sensation, headache, dizziness, or nausea during the intervention.

**Table 2:** Mean stimulation intensity and side effects under each condition (mean ± standard deviation).

Condition	Stimulus intensity (mA)	Itching (0–6)
taVNS	1.45 ± 0.27	2.77 ± 0.81
sham	1.75 ± 0.36	2.55 ± 0.74

0 = “no sensation,” 6 = “very strong sensation”.

taVNS, transcutaneous auricular vagus nerve stimulation.

### 3.2 sAA, GoRT, and SSRT under each stimulation condition

**Figure 3:**
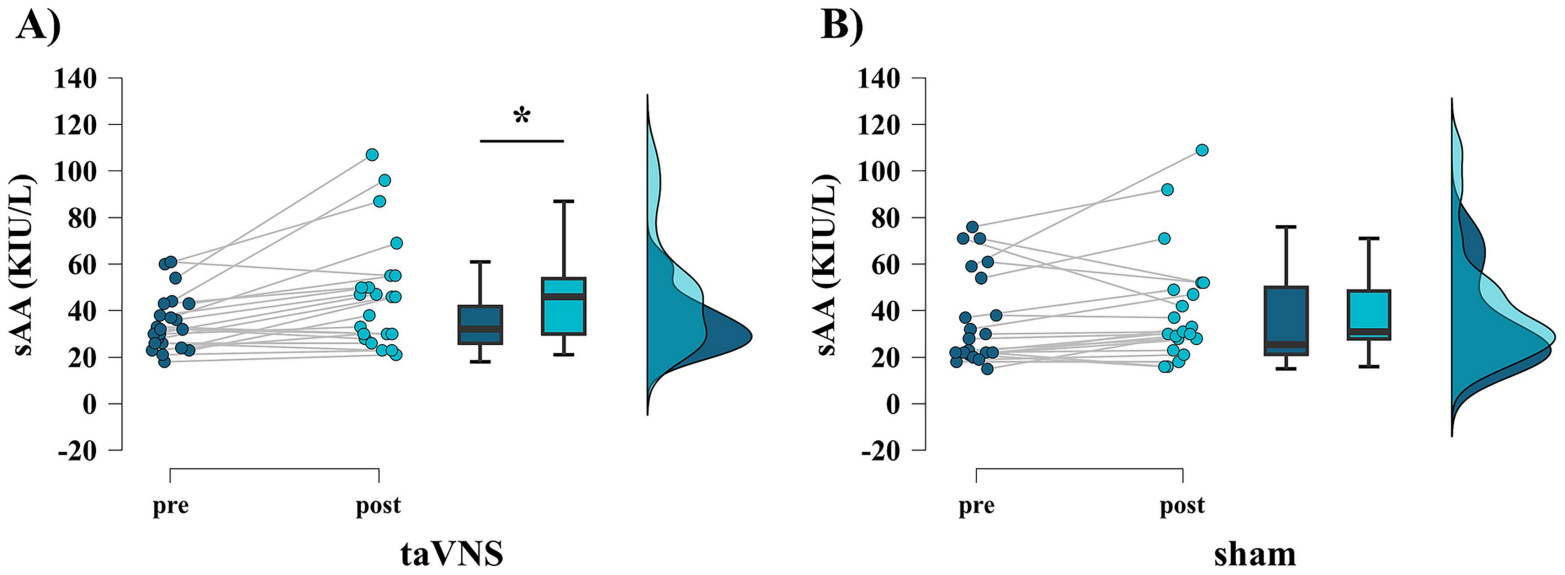
Results of salivary alpha-amylase (sAA) levels pre- and poststimulation for each condition. **(A)** taVNS condition. **(B)** Sham condition. (*p < 0.05).

**Figure 4:**
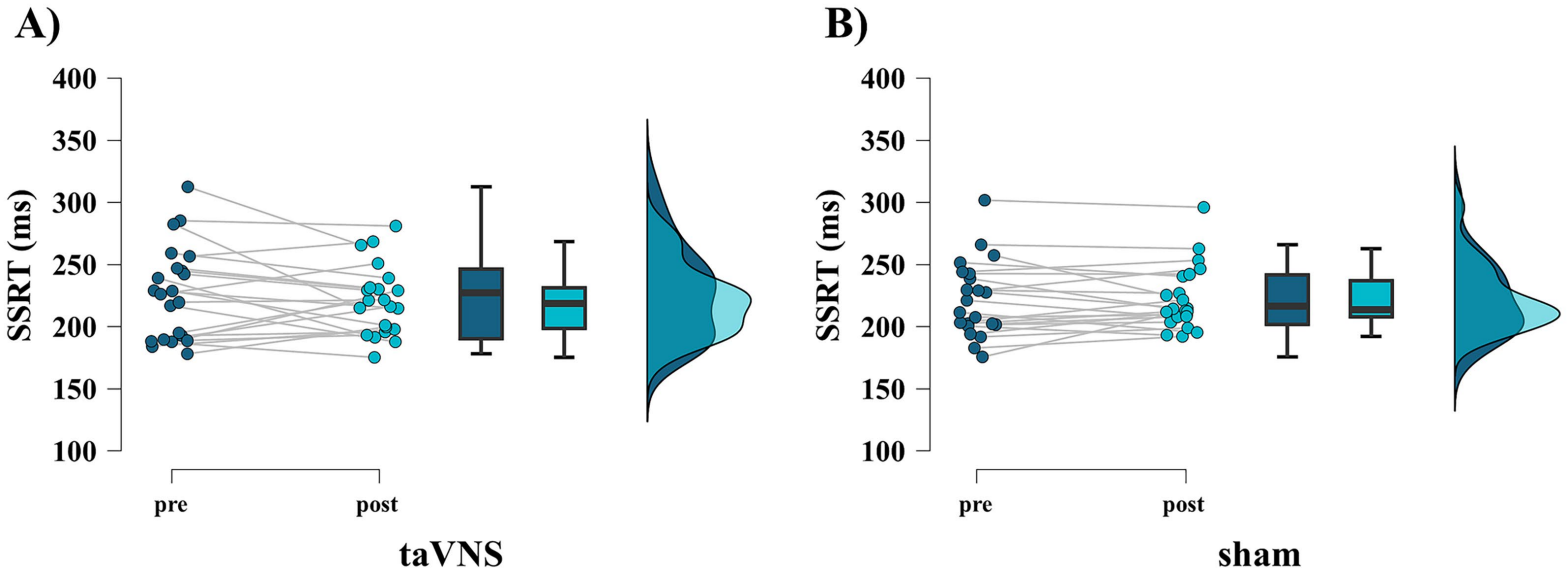
Results of the stop signal reaction time (SSRT) pre- and poststimulation for each condition. **(A)** taVNS condition. **(B)** Sham condition.

Table 3 presents the pre- and post-sAA, GoRT, SSD, p(response∣signal), and SSRT results for each stimulation condition. sAA levels were significantly higher after stimulation than before stimulation only under the taVNS condition (taVNS: *p* = 0.002, *r* = 0.668, Figure 3A; sham: *p* = 0.092, *r* = 0.360, Figure 3B). Meanwhile, no significant difference was observed in GoRT between before and after stimulation under either condition (taVNS: *p* = 0.465, *r* = 0.156; sham: *p* = 0.168, *r* = 0.294). Similarly, no significant difference was observed in SSD between before and after stimulation under either condition (taVNS: *p* = 0.189, *r* = 0.280; sham: *p* = 0.147, *r* = 0.309). In addition, no significant difference was observed in the probability of responding to the stop signal, p(response∣signal), between before and after stimulation under either condition (taVNS: *p* = 0.058, *r* = 0.405; sham: *p* = 0.686, *r* = 0.086). Concerning SSRT, repeated-measures two-way ANOVA revealed no main effect of time factor (*F*_(1, 21)_ = 0.703, *p* = 0.411, *η*^2^*p* = 0.032) or stimulation factor (*F*_(1, 21)_ = 0.106, *p* = 0.748, *η*^2^*p* = 0.005), and no interaction was observed (*F*_(1, 21)_ = 1.091, *p* = 0.308, *η*^2^*p* = 0.049, Figures 4A,B). To complement the frequentist approach, a Bayesian repeated-measures ANOVA was also conducted. This analysis revealed that the alternative models, including stimulation (BF_10_ = 0.277), time (BF_10_ = 0.272), and their interaction (BF_10_ = 0.140), were less supported than the null model. These results provide moderate-to-strong Bayesian evidence in favor of the null hypothesis, suggesting the absence of these effects.

**Table 3:** Measured values of sAA, SSRT, and GoRT before and after the intervention.

Condition	sAA (KIU/L)		GoRT (ms)		SSD (ms)		p(response|sigunal)		SSRT (ms)	
	Pre	Post	Pre	Post	Pre	Post	Pre	Post	Pre	Post
taVNS	32.0 (25.5–43.0)	46.0 (29.5–55.0)	368.6 (357.7–405.2)	380.5 (347.2–412.9)	133.0 (107.4–191.4)	153.4 (104.0–186.2)	50.9 (49.1–54.5)	50.0 (49.1–51.6)	227.0 ± 37.3	220.7 ± 27.9
Sham	25.5 (21.0–55.3)	31.0 (26.8–49.8)	378.3 (345.1–430.5)	378.0 (356.9–468.8)	161.3 (102.5–204.9)	153.7 (119.9–236.7)	50.0 (49.1–54.9)	50.0 (48.4–50.9)	221.9 ± 30.4	222.3 ± 25.6

sAA, salivary alpha-amylase; GoRT, go reaction time; SSD, stop signal delay; p(response|sigunal), probability (response∣signal); SSRT, stop signal reaction time.

The results for sAA, GoRT, SSD and p(response|sigunal) are presented as the median (interquartile range).

The results for SSRT are displayed as the mean ± standard deviation.

### 3.3 Correlation between pre_SSRT and ΔSSRT under each condition

**Figure 5:**
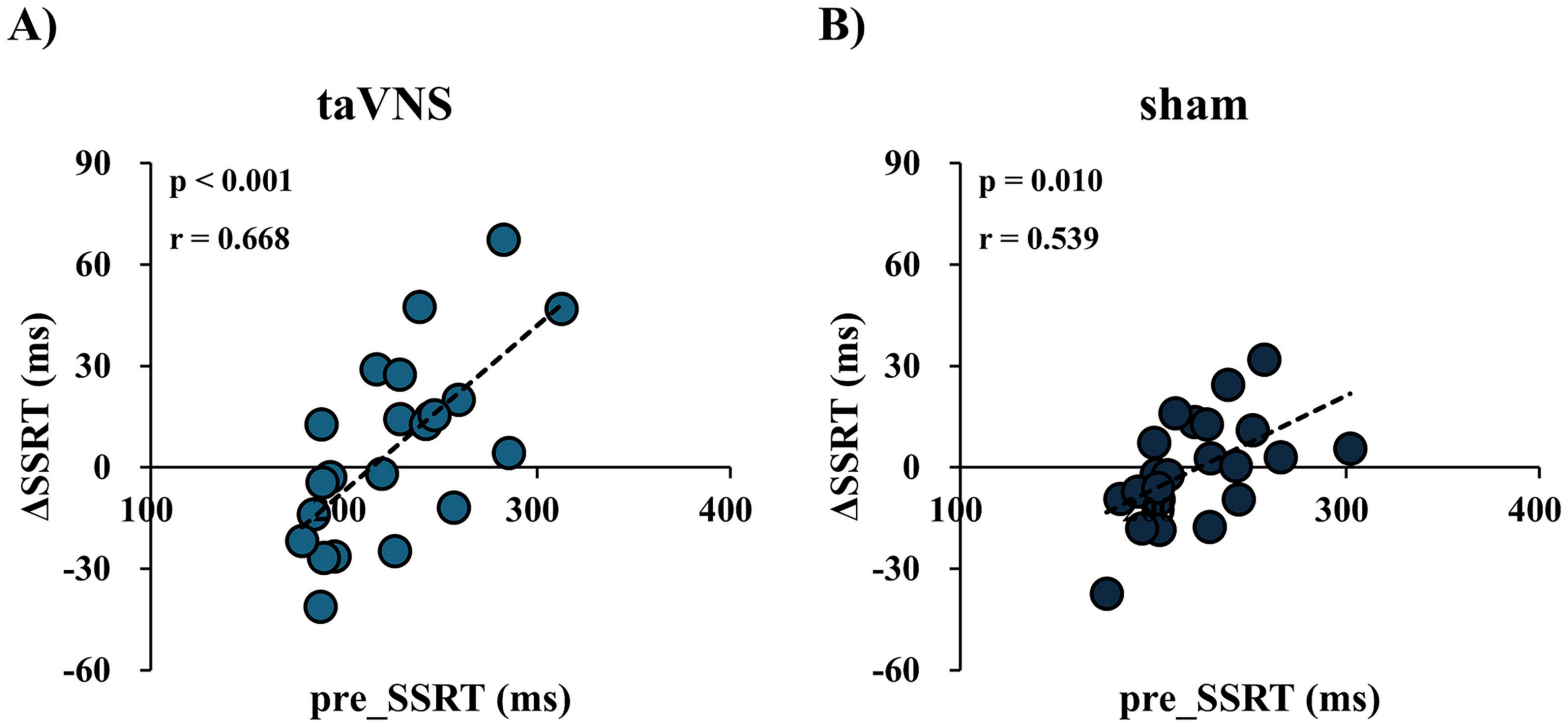
Correlation between prestimulus stop signal reaction time (pre_SSRT) and the change in the stop signal reaction time between before and after the intervention (ΔSSRT) under each condition. **(A)** Transcutaneous auricular vagus nerve stimulation (taVNS) condition. **(B)** Sham condition.

As presented in Figure 5, pre_SSRT and ΔSSRT were positively correlated under both the taVNS and sham conditions (taVNS condition: *p* < 0.001, Pearson’s *r* = 0.668, Figure 5A; sham condition: *p* = 0.010, Pearson’s *r* = 0.539, Figure 5B).

### 3.4 ECG

**Figure 6:**
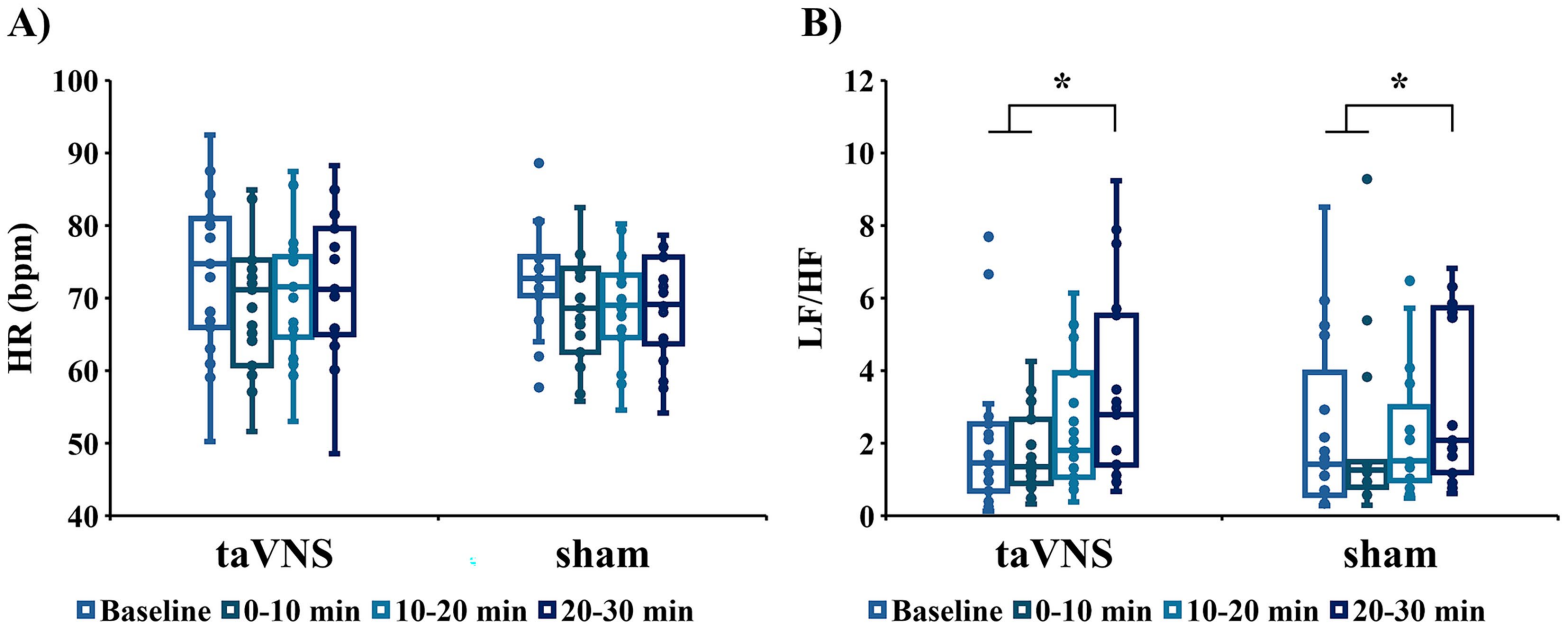
Heart rate (HR) and the low-frequency component/high-frequency component ratio (LF/HF) at baseline, 0–10 min, 10–20 min, and 20–30 min under each condition: **(A)** HR; **(B)** LF/HF (*p < 0.05).

Figure 6 presents the HR and LF/HF results under each condition. Regarding HR, repeated-measures two-way ANOVA revealed a main effect of the time factor (*F*_(3, 54)_ = 20.914, *p* < 0.001, *η*^2^*p* = 0.537) but no main effect of the stimulus factor (*F*_(1, 18)_ = 0.494, *p* = 0.491, *η*^2^*p* = 0.027) and no interaction (*F*_(3, 54)_ = 2.566, *p* = 0.064, *η*^2^*p* = 0.125, Figure 6A). Significant differences in LF/HF were observed under both the taVNS and sham conditions (taVNS condition: *p* < 0.001; sham condition: *p* = 0.009, Figure 6B). *Post hoc* tests illustrated that under the taVNS condition, LF/HF 20–30 min after the start of stimulation was significantly higher than that at baseline (*p* = 0.001) and 0–10 min after the start of stimulation (*p* = 0.004). Even in the sham condition, LF/HF 20–30 min after the start of stimulation was significantly higher than baseline (*p* = 0.034) and 0–10 min after the start of stimulation (*p* = 0.023).

## 4 Discussion

This study aimed to clarify the effects of taVNS-induced NA modulation on response inhibition under a specific stimulation protocol in healthy male participants. The results showed that, although intermittent taVNS for 30 min was associated with an increase in sAA—suggesting enhanced NA activity—no corresponding improvement in response inhibition was observed. Moreover, a significant positive correlation was observed between pre_SSRT and ΔSSRT under both conditions, indicating that participants with a longer pre_SSRT exhibited a greater reduction in SSRT. This association is correlational in nature and was observed irrespective of stimulation conditions, suggesting that it may reflect a general relationship between baseline performance and subsequent change rather than a taVNS-specific effect. This finding should therefore be interpreted with caution, as alternative explanations, such as regression to the mean, cannot be ruled out.

In the present study, stimulation intensity was significantly higher in the sham condition than in the taVNS condition. Stimulation intensity was individually determined for each participant using the same titration procedure in both conditions. Previous studies have similarly reported that sham stimulation of the earlobe tends to require higher stimulation intensities than taVNS applied to the concha, although these differences were not statistically significant (Horinouchi et al., 2024; Ventura-Bort et al., 2018). The ABVN, which is involved in somatosensory processing, is most densely distributed in the cymba concha but sparsely innervates the earlobe, as demonstrated by human anatomical studies (Peuker and Filler, 2002). Accordingly, higher current intensities may have been necessary in the sham condition to elicit subjective sensations comparable to those experienced during taVNS.

sAA is secreted in response to sympathetic nervous system activity (Bosch et al., 2011), and pharmacological studies suggest that it may reflect, at least in part, central NA activity (Ehlert et al., 2006; Warren et al., 2017). taVNS is thought to engage the NTS via the auricular branch of the vagus nerve and may indirectly influence the LC–NA system through projections from the NTS (Frangos et al., 2015; Nemeroff et al., 2006; Yakunina et al., 2017). LC is the primary source of NA in the central nervous system (Ishimatsu and Williams, 1996; Hachem et al., 2018). Previous studies have reported increases in sAA following taVNS, suggesting that these changes may be associated with modulation of the LC–NA system and enhanced noradrenergic activity (Ventura-Bort et al., 2018; Warren et al., 2019). Consistent with these findings, taVNS in the present study may have indirectly influenced the LC–NA system via NTS projections, resulting in changes in central NA activity and concomitant increases in sAA levels. However, the interpretative specificity of sAA remains limited. Although sAA is frequently used as an indirect marker of LC–NA activity, it may more broadly reflect sympathetic activation and global autonomic arousal rather than LC-specific modulation of inhibitory-control networks (Burger et al., 2020). Therefore, the increase in sAA observed in the present study should be interpreted cautiously as being compatible with, but not definitive evidence for, modulation of central noradrenergic processes via the NTS–LC pathway.

Despite the observed increase in sAA levels, taVNS did not improve response inhibition in this study. One possible reason for this result is that NA levels were insufficiently increased by taVNS. Previous neuroimaging studies investigated the brain regions involved in reactive response inhibition, revealing the primary involvement of frontal regions, including the right inferior frontal gyrus (rIFG), presupplementary motor area, and dorsolateral prefrontal cortex (Friehs et al., 2021; Hughes et al., 2013; Swann et al., 2012, 2013). In an fMRI study, rIFG activation was increased by the administration of atomoxetine, which enhances central NA levels (Chamberlain et al., 2009). Furthermore, the effect of atomoxetine on rIFG activation during successful inhibition increased in a plasma concentration-dependent manner (Chamberlain et al., 2009). This suggests that the enhancement of the NA system in key brain regions contributes to response inhibition performance. However, although sAA is widely used as an indirect marker of NA activity, its fluctuations are influenced by global sympathetic nervous system activation and therefore do not necessarily reflect region-specific NA modulation within brain areas involved in response inhibition, such as the IFG. Consequently, while the increase in sAA observed in the present study may suggest an elevation in central NA activity, it does not guarantee a functionally relevant or region-specific increase in NA within the response inhibition network. Moreover, although the changes in sAA levels in this study are consistent with increased central NA activity, their magnitude appears to be modest compared with those reported in studies using atomoxetine. This difference may be attributable to the distinct mechanisms of action of taVNS and atomoxetine. Evidence from clinical studies in children, adolescents, and adults suggests that atomoxetine increases NA concentrations in the synaptic cleft by inhibiting presynaptic NA transporters and thereby preventing reuptake (Wilens, 2006). In addition, human studies have reported that this pharmacologically induced increase in NA reaches a plateau approximately 90 min after administration and persists for up to 9 hours (Sauer et al., 2005). In contrast, taVNS has been shown to induce activation of the LC following stimulation (Frangos et al., 2015; Yakunina et al., 2017), and such LC activation is thought to facilitate noradrenergic projections to widespread regions of the central nervous system (Benarroch, 2018; Hachem et al., 2018; Ma et al., 2023). However, to the best of our knowledge, there is currently no evidence from human studies that taVNS directly affects NA transporters. Accordingly, any increase in NA associated with taVNS may be more susceptible to reuptake and therefore more transient compared with the effects induced by atomoxetine. Furthermore, taVNS in rats has been reported to increase extracellular NA during stimulation; however, the NA levels decrease within 1 h after the end of the stimulation (Roosevelt et al., 2006). However, it remains unclear whether taVNS elicits similar effects in humans. Although the precise timing and extent of sAA level reduction remain unknown, the increase in NA levels observed in this study may not have activated brain regions, such as the rIFG, potentially because of the reuptake mechanisms or suppressed release following stimulation and may have been insufficient to enhance response inhibition. Additionally, although Ventura-Bort et al. (2018) employed continuous stimulation, the present study adopted intermittent stimulation with a 30-s on/off cycle, as implemented in commercially available stimulators, consistent with the approach used by Warren et al. (2019). This difference in stimulation cycle may have influenced the increase in NA levels. Horinouchi et al. (2024) reported that functions related to short-interval intracortical inhibition, including facilitation of cholinergic circuits, were enhanced only under continuous stimulation conditions, whereas no comparable effects were observed with intermittent stimulation consisting of 30 s on/30 s off cycles. This finding suggests that the duty cycle of stimulation may play a critical role in determining neuromodulatory outcomes. In fact, despite the comparable stimulation intensity, the sAA levels were lower in the present study than in the study by Ventura-Bort et al. (2018). These findings indicate that the intermittent stimulation adopted in this study may not have induced sufficient NA release to enhance response inhibition following stimulation. Previous studies have reported that taVNS administered during cognitive or inhibitory tasks improves task performance, indicating that NA regulation during taVNS contributes to such improvements (Jacobs et al., 2015; Beste et al., 2016; Fischer et al., 2018; Jongkees et al., 2018). As mentioned above, considering the possibility of NA reuptake and release inhibition, performing tasks during, rather than after, taVNS may effectively enhance cognitive and inhibitory functions. However, the present study focused exclusively on post-stimulation effects and did not directly compare the effects of taVNS administered during task performance with those observed after stimulation. Consequently, it remains unclear which timing of taVNS administration constitutes the more effective intervention strategy. Moreover, the existing taVNS literature is characterized by substantial heterogeneity in stimulation protocols, including stimulation site, laterality, stimulation pattern (e.g., continuous vs. intermittent), and stimulation cycles, and studies reporting performance improvements during taVNS have employed diverse combinations of these parameters. Therefore, future research is needed to systematically identify both the optimal stimulation parameters and the most effective timing of intervention.

Another possible explanation relates to the characteristics of the task employed in the present study. The stop-signal task used here involves relatively simple motor responses and limited response-selection demands. Previous research has suggested that inhibitory control is influenced not only by conflict processing but also by movement complexity and action-selection requirements (Gálvez-García et al., 2018). In addition, recent studies indicate that neuromodulatory effects, including those of taVNS, may become more pronounced under conditions of increased task complexity and higher executive demands (Mena-Chamorro et al., 2025; Gálvez-García et al., 2026). For example, previous research has shown that taVNS-related improvements in inhibitory control become evident particularly under conditions of increased working memory load, suggesting that the effects of taVNS may depend on the level of cognitive demand imposed by the task (Beste et al., 2016). Therefore, the absence of behavioral effects in the present study may not solely reflect insufficient noradrenergic modulation but may also be attributable to the relatively low cognitive and motor demands of the task employed.

In the present study, we focused on NA regulation to examine the effects of taVNS; however, accumulating evidence suggests that the neuromodulatory effects of taVNS may not be limited to the NA system alone. Review articles have indicated that taVNS may also influence other neurotransmitter systems, including the GABAergic and cholinergic systems (Colzato and Beste, 2020). Furthermore, studies in healthy young adults have reported associations between individual differences in GABAergic function and SST performance (Paci et al., 2021). Importantly, recent evidence suggests that noradrenergic and GABAergic systems may interact to regulate neural gain and inhibitory processing, thereby shaping cognitive control performance (Keute et al., 2018). Taken together, these findings raise the possibility that the balance and interaction between NA and GABAergic mechanisms, rather than NA modulation alone, may have contributed to the present results. However, because the present study did not include measures that directly index other neurotransmitter systems, including GABAergic function, caution is warranted in attributing the neuromodulatory effects of taVNS exclusively to the NA system.

Another possible explanation is that practice and habituation effects cannot be fully ruled out in the present study, as participants completed the SST repeatedly across pre- and post-intervention assessments in two separate sessions. Although the order of conditions was counterbalanced and a one-week washout period was implemented, repeated exposure to the task and laboratory context may have contributed to improvements in behavioral performance independently of taVNS. Such practice-related improvements may have attenuated both behavioral and autonomic responses, thereby reducing sensitivity to detect taVNS-specific effects.

A significant positive correlation was observed between pre_SSRT and ΔSSRT in this study. Notably, this association was present under both the taVNS and sham conditions, suggesting that it is unlikely to reflect a stimulation-specific effect. One possible explanation is that participants with longer pre_SSRTs had greater potential for improvement and therefore exhibited larger reductions in SSRT with repeated task exposure, whereas those with shorter pre_SSRTs may have shown limited improvement due to ceiling effects. Baseline variability (pre_SSRT) was comparable between conditions (see Table 3). Therefore, the observed correlation is unlikely to be explained by differences in baseline variability. Taken together, these findings suggest that the association reflects non-specific factors, such as practice-related improvements, rather than taVNS-induced modulation of inhibitory control.

As taVNS has also been reported to increase parasympathetic nerve activity, ECGs were measured in this study. However, the stimulation did not affect HR. Previous studies conducted by our research group found that a stimulation intensity of 3.0 mA is required to change HR (Yokota et al., 2022), whereas the stimulation intensity in the present study was approximately 1.5 mA, suggesting that it was insufficient to modulate HR. Based on the LF/HF results, sympathetic activity gradually increased over time under both stimulation conditions, with no condition-specific effects of taVNS observed. Although many previous studies have reported LF/HF fluctuations following taVNS, relatively few have examined changes during stimulation. The present findings may therefore reflect the prolonged stimulation duration (30 min), which could have contributed to a time-dependent increase in sympathetic activity. Importantly, the gradual increase in the LF/HF ratio observed in both conditions raises questions regarding the physiological inertness of the sham condition. In the present study, the sham condition involved a higher stimulation intensity than the taVNS condition, which may have inadvertently engaged somatosensory afferent pathways and elicited nonspecific autonomic and arousal-related responses. Notably, prior work indicates that earlobe stimulation, despite lacking canonical vagal innervation, can nonetheless influence somatosensory and autonomic processes (Burger et al., 2020; Kraus et al., 2013). Such nonspecific effects may have attenuated the contrast between conditions, thereby contributing to the absence of significant differences in both physiological and behavioral outcomes. Future studies should therefore carefully optimize sham stimulation protocols, including the matching of stimulation intensity across conditions, and systematically assess the extent to which nonspecific sensory and arousal-related effects shape autonomic and cognitive responses.

This study had several limitations. First, an important consideration relates to statistical power. The sample size was determined based on an *a priori* power analysis assuming a medium effect size (*f* = 0.25), which is common in experimental research. However, behavioral effects of taVNS on executive functions are often small to moderate and may exhibit substantial interindividual variability. Therefore, the present study was primarily powered to detect medium-sized effects, and smaller but potentially meaningful effects may not have been detected. This limitation should be considered when interpreting the null findings. Second, sAA is merely an indirect indicator, it is uncertain whether central NA levels changed. Although it has often been used as a proxy of central NA activity, its specificity as an index of LC–NA function remains debated, because it may also reflect broader sympathetic activation and general arousal rather than LC-specific neural modulation (Burger et al., 2020). Therefore, the increase in sAA observed after taVNS should not be interpreted as direct evidence that central NA levels, or LC activity specifically, changed. In addition to sAA, indicators for evaluating central NA include salivary cortisol, pupillary response, and the P3 component of event-related brain potentials, all of which are also used in taVNS research (Ventura-Bort et al., 2018; Warren et al., 2019; Capone et al., 2021). Therefore, these evaluation indicators might have also been influenced by the stimulation. In the future, a more detailed evaluation of central NA may be achieved through a simultaneous measurement of sAA and these additional indicators. Moreover, sAA is sensitive to circadian variation, which may complicate its interpretation, particularly when relying on short-term measurements. Accordingly, future research should consider strategies to mitigate the influence of diurnal fluctuations and incorporate alternative or complementary biomarkers to provide a more robust assessment of noradrenergic activity. Moreover, sAA measurements were not performed before or after the SST. Therefore, it remains unclear how the sAA levels, which increased after taVNS, changed following the SST. As aforementioned, a decrease in sAA levels may have occurred if NA reuptake or release was inhibited. Furthermore, the present study did not examine the association between changes in sAA and changes in SSRT. Consequently, it remains unclear whether the observed null behavioral effects reflect insufficient neuromodulation, limited behavioral sensitivity of the task, or a dissociation between peripheral indicators and central noradrenergic activity. Taken together, these limitations suggest that future studies should adopt a more comprehensive evaluation of central NA function, incorporating dose–response relationships as well as correlational analyses between neurophysiological indices and behavioral measures. Third, because fMRI was not conducted in this study, it remains unclear whether the LC, which is the source of NA, or the brain regions involved in response inhibition were activated. In future research, in addition to identifying the optimal stimulation parameters, it will be necessary to measure multiple NA evaluation indicators and assess brain activity using fMRI. Fourth, only male participants were included in this study. This sampling approach was intended to minimize potential variability and enhance experimental control; however, it inherently reflects a trade-off with external validity and limits the generalizability of the findings to female populations. Restricting the sample to males may also introduce systematic sampling bias and complicate the interpretation of the observed null effects, as potential sex-related differences in the effects of taVNS on noradrenergic regulation and response inhibition cannot be excluded. Therefore, the findings of this study should be interpreted as specific to healthy male participants, and caution should be exercised against overgeneralization. To the best of our knowledge, this is the first study to investigate how NA regulation following taVNS affects response inhibition. By conducting research that addresses these limitations and challenges, it might be possible to clarify the relationship between NA regulation by taVNS and response inhibition, and our findings will serve as a foundation for future studies.

## 5 Conclusion

In healthy male participants, no evidence for an improvement in response inhibition was observed under the specific stimulation protocol and task conditions employed in the present study. These findings should therefore be interpreted within the boundary conditions of this experimental paradigm, rather than as evidence for a general absence of taVNS effects on inhibitory control. Given the substantial heterogeneity of stimulation parameters across the taVNS literature, including stimulation site, laterality, and stimulation pattern, such protocol characteristics should be considered central to the interpretation of behavioral outcomes. Future studies should systematically examine how variations in stimulation parameters influence noradrenergic and related neuromodulatory processes, incorporate multimodal neurophysiological measures, and include both male and female participants in line with current recommendations to consider sex as a biological variable in human research, to improve generalizability.

## Data Availability

The raw data supporting the conclusions of this article will be made available by the authors, without undue reservation.
